# Draft Genome Sequence of the Toxic Bloom-Forming Cyanobacterium *Aphanizomenon flos-aquae* NIES-81

**DOI:** 10.1128/genomeA.00044-14

**Published:** 2014-02-13

**Authors:** Huansheng Cao, Yohei Shimura, Kawachi Masanobu, Yanbin Yin

**Affiliations:** a Department of Biological Sciences, Northern Illinois University, DeKalb, Illinois, USA; b Microbial Culture Collection, National Institute for Environmental Studies, Tsukuba, Ibaraki, Japan

## Abstract

*Aphanizomenon flos-aquae* is a toxic filamentous cyanobacterium that causes water blooms in freshwaters across the globe. We present the draft genome sequence of the *A. flos-aquae* strain NIES-81, which was determined by 454 pyrosequencing technology. The draft genome is ~5.7 Mb, containing 5,802 predicted protein-coding genes and 58 RNA genes, with a G+C content of 38.5%.

## GENOME ANNOUNCEMENT

Cyanobacterial blooms have arisen with increasing frequency and impact over the last few decades. Among the top bloomers, *Aphanizomenon flos-aquae* is a toxic filamentous species that forms blooms around the world (1). Although its ecophysiology for bloom formation has been studied extensively (2), further insights into its population dynamics, molecular physiology, and ecology will greatly benefit from the sequencing of its genome, as is the case for other bloomers, such as *Microcystis aeruginosa* (3) and *Anabaena* spp. (4).

The clonal axenic strain NIES-81 was isolated from Lake Kasumigaura, Japan, in 1978 and cryopreserved at the Microbial Culture Collection of the National Institute for Environmental Studies of Japan. The genomic DNA was extracted using an enzymatic cell lysis method (5). The complete genome sequence of NIES-81 was determined by a combination of single-end and paired-end sequencing by 454 pyrosequencing technology on a GS FLX Titanium platform (Roche, Inc.), with a sequencing depth of ~79×. The reads were assembled using the GS *de novo* Assembler 2.9; genes contained in the draft genome were annotated using the RAST Web server (6), which predicted both protein- and RNA-coding genes. Protein functions were also inferred and classified by running homology searches against the COG database (7), the InterPro database (8), and the NCBI nonredundant protein database.

A total of 706,989 single-end and 140,948 paired-end reads were obtained from shotgun libraries. The average read lengths are 575 and 436 bases for each library. The overall G+C content of the assembled draft genome is 38.5%. It contains 5,682,503,404 bases in 3 large and 110 smaller scaffolds. The draft genome contains 5,802 predicted protein-coding sequences, 42 tRNA genes, and 16 rRNA genes. Similar to the genomes of *M. aeruginosa* (3) and *Anabaena* spp. (4), the *A. flos-aquae* draft genome is most enriched with genes involved in amino acid (251) and carbohydrate (223) metabolic functions.

### Nucleotide sequence accession number.

This genome sequence has been deposited at GenBank with the accession no. AZYY00000000.
